# Integrative genomics for mango genetics and breeding

**DOI:** 10.1093/hr/uhaf260

**Published:** 2025-09-24

**Authors:** Bilal Ahmad, Ying Su, Rida Arshad, Tayyaba Razzaq, Yi Zhang, Ting Hou, Chaochao Li, Zhongxin Jin, Chengjie Chen, Peng Wang, Melanie J Wilkinson, Yibo Bai, Yeyuan Chen, Yu Zhang, Zhiguo Dang, Yongfeng Zhou, Xinmin Tian, Jianfeng Huang

**Affiliations:** State Key Laboratory of Tropical Crop Breeding, Tropical Crops Genetic Resources Institute, Chinese Academy of Tropical Agricultural Sciences, Haikou, Hainan 571101, China; State Key Laboratory of Tropical Crop Breeding, Shenzhen Branch, Guangdong Laboratory of Lingnan Modern Agriculture, Key Laboratory of Synthetic Biology, Ministry of Agriculture and Rural Affairs, Agricultural Genomics Institute at Shenzhen, Chinese Academy of Agricultural Sciences, Shenzhen, China; State Key Laboratory of Tropical Crop Breeding, Tropical Crops Genetic Resources Institute, Chinese Academy of Tropical Agricultural Sciences, Haikou, Hainan 571101, China; State Key Laboratory of Tropical Crop Breeding, Shenzhen Branch, Guangdong Laboratory of Lingnan Modern Agriculture, Key Laboratory of Synthetic Biology, Ministry of Agriculture and Rural Affairs, Agricultural Genomics Institute at Shenzhen, Chinese Academy of Agricultural Sciences, Shenzhen, China; State Key Laboratory of Tropical Crop Breeding, Tropical Crops Genetic Resources Institute, Chinese Academy of Tropical Agricultural Sciences, Haikou, Hainan 571101, China; State Key Laboratory of Tropical Crop Breeding, Shenzhen Branch, Guangdong Laboratory of Lingnan Modern Agriculture, Key Laboratory of Synthetic Biology, Ministry of Agriculture and Rural Affairs, Agricultural Genomics Institute at Shenzhen, Chinese Academy of Agricultural Sciences, Shenzhen, China; State Key Laboratory of Tropical Crop Breeding, Tropical Crops Genetic Resources Institute, Chinese Academy of Tropical Agricultural Sciences, Haikou, Hainan 571101, China; State Key Laboratory of Tropical Crop Breeding, Shenzhen Branch, Guangdong Laboratory of Lingnan Modern Agriculture, Key Laboratory of Synthetic Biology, Ministry of Agriculture and Rural Affairs, Agricultural Genomics Institute at Shenzhen, Chinese Academy of Agricultural Sciences, Shenzhen, China; State Key Laboratory of Tropical Crop Breeding, Tropical Crops Genetic Resources Institute, Chinese Academy of Tropical Agricultural Sciences, Haikou, Hainan 571101, China; State Key Laboratory of Tropical Crop Breeding, Shenzhen Branch, Guangdong Laboratory of Lingnan Modern Agriculture, Key Laboratory of Synthetic Biology, Ministry of Agriculture and Rural Affairs, Agricultural Genomics Institute at Shenzhen, Chinese Academy of Agricultural Sciences, Shenzhen, China; State Key Laboratory of Tropical Crop Breeding, Shenzhen Branch, Guangdong Laboratory of Lingnan Modern Agriculture, Key Laboratory of Synthetic Biology, Ministry of Agriculture and Rural Affairs, Agricultural Genomics Institute at Shenzhen, Chinese Academy of Agricultural Sciences, Shenzhen, China; State Key Laboratory of Tropical Crop Breeding, Tropical Crops Genetic Resources Institute, Chinese Academy of Tropical Agricultural Sciences, Haikou, Hainan 571101, China; State Key Laboratory of Tropical Crop Breeding, Shenzhen Branch, Guangdong Laboratory of Lingnan Modern Agriculture, Key Laboratory of Synthetic Biology, Ministry of Agriculture and Rural Affairs, Agricultural Genomics Institute at Shenzhen, Chinese Academy of Agricultural Sciences, Shenzhen, China; State Key Laboratory of Tropical Crop Breeding, Tropical Crops Genetic Resources Institute, Chinese Academy of Tropical Agricultural Sciences, Haikou, Hainan 571101, China; State Key Laboratory of Tropical Crop Breeding, Tropical Crops Genetic Resources Institute, Chinese Academy of Tropical Agricultural Sciences, Haikou, Hainan 571101, China; State Key Laboratory of Tropical Crop Breeding, Tropical Crops Genetic Resources Institute, Chinese Academy of Tropical Agricultural Sciences, Haikou, Hainan 571101, China; School of the Environment, The University of Queensland, Brisbane, Qld 4072, Australia; Australian Research Council Centre of Excellence for Plant Success in Nature and Agriculture, The University of Queensland, Brisbane, Qld 4072, Australia; State Key Laboratory of Tropical Crop Breeding, Tropical Crops Genetic Resources Institute, Chinese Academy of Tropical Agricultural Sciences, Haikou, Hainan 571101, China; Sanya Research Institute of Chinese Academy of Tropical Agricultural Sciences, Sanya 572025, China; Guangxi Subtropical Research Institute, Guangxi Academy of Agricultural Sciences, Nanning 530001, China; State Key Laboratory of Tropical Crop Breeding, Tropical Crops Genetic Resources Institute, Chinese Academy of Tropical Agricultural Sciences, Haikou, Hainan 571101, China; State Key Laboratory of Tropical Crop Breeding, Tropical Crops Genetic Resources Institute, Chinese Academy of Tropical Agricultural Sciences, Haikou, Hainan 571101, China; State Key Laboratory of Tropical Crop Breeding, Shenzhen Branch, Guangdong Laboratory of Lingnan Modern Agriculture, Key Laboratory of Synthetic Biology, Ministry of Agriculture and Rural Affairs, Agricultural Genomics Institute at Shenzhen, Chinese Academy of Agricultural Sciences, Shenzhen, China; Key Laboratory of Ecology of Rare and Endangered Species and Environmental Protection (Ministry of Education) & Guangxi Key Laboratory of Landscape Resources Conservation and Sustainable Utilization in Lijiang River Basin, Guangxi University Engineering Research Center of Bioinformation and Genetic Improvement of Specialty Crops, Guangxi 541006, China; State Key Laboratory of Tropical Crop Breeding, Tropical Crops Genetic Resources Institute, Chinese Academy of Tropical Agricultural Sciences, Haikou, Hainan 571101, China

## Abstract

Mango is the second most important tropical fruit crop. Due to ever-changing environmental conditions, world mango production is facing challenges such as diseases (anthracnose and mango malformation), physiological disorders (alternate bearing), low fruit setting, poor fruit quality, short shelf life, and climate change adaptation. Breeding efforts are hindered by the long juvenile period, outdated breeding system, and high heterozygosity, resulting in a slow pace of mango improvement programs. However, over the last decade, significant advances in high-quality genome assemblies, pangenomics, genetic mapping, multiomics data, and phenomics of large populations have accelerated crop genetics and breeding. Here, we summarize recent progress on the origin and domestication of mango, advancements in genome assemblies, development of genetic maps, functional and comparative genomics, evolutionary insights, and assessments of global phenotypic and genotypic diversity, including species at risk. We also discuss the integration of multiomics approaches with quantitative genetics for crop improvement. Furthermore, we highlight the key research gaps that limit breeding efficiency and propose integrative strategies combining pangenomics, multiomics, and machine learning with improved transformation protocols and multienvironment testing to accelerate the development of climate-resilient, high-quality mango cultivars.

## Introduction

Mango (*Mangifera indica* L.) is a member of the family Anacardiaceae, and the genus *Mangifera*, which includes 69 species, 27 of which produce edible fruits [1, 2]. It is well known as the ‘king of fruits’ for its attractive aroma, appearance, high nutrition, and delicious taste [3]. It is widely cultivated in tropical and warmer subtropical regions [3, 4]. It is grown across all continents (excluding Antarctica) and in >103 countries. In 2021, global mango production exceeded 51 million metric tons (Mt). India is the leading producer, followed by China. Interestingly, Mexico is fourth largest producer but a leading exporter of mango [5]. Mango is a highly heterozygous and cross-pollinated fruit plant [2].

Based on the origin, mangoes are classified into two primary cultivar types: Indian and Indochinese. Indian cultivars have a distinct color change when ripe, transitioning to shades of orange or red. They also have rounded shapes, strong-flavored fibrous flesh, and monoembryonic seeds. Unlike Indian cultivars, Indochinese mangoes have polyembryonic seeds, which contain a single zygotic embryo and multiple embryos derived from maternal nucellar tissue [1]. This nucellar embryony trait is uncommon among angiosperms; however, it has been reported in three other Mangifera species: *M. laurina*, *M. odorata*, and *M. casturi* [6, 7]. The majority of polyembryonic fruits have a green to yellow pericarp, with the Thai mango (*M. siamensis* warbg. ex Craib) being the most common example [8–10]. The monoembryonic cultivars hold significant commercial importance in Africa, India, Florida, and South America due to their superior fruit quality characteristics. On the other hand, polyembryonic mango cultivars are favored in Australia, Central America, Haiti, Southeast Asia, Hawaii, and South Africa [11, 12].

The domestication and selection of mango varieties has a rich history spanning thousands of years. Traditional varieties have generally been developed through vegetative propagation by grafting mutant branches. Conventional breeding is not new for mango but became popular after its introduction in the USA, Australia, and China [13]. New fruit cultivars with improved and desirable characteristics have been developed through traditional breeding. However, traditional breeding is unsuitable for developing new desirable mango cultivars due to polyembryony, heterozygosity, long juvenility, more fruit drop (low fruit setting), and long breeding cycles [14]. Integrative genomics is required to accelerate mango breeding progress.

Initially, mango was considered an allotetraploid plant with a genome size of 450 Mb [15, 16]. However, several authors [9, 17, 18] have reported that genetic markers for mango are inherited in a disomic manner, indicating that mango may be better treated as a diploid (two complete sets of chromosomes one from each parent). Mango has 2*n* = 2*x* = 40 chromosomes, suggesting that there are 20 haploid chromosomes and 20 linkage groups [1].

**Figure 1:**
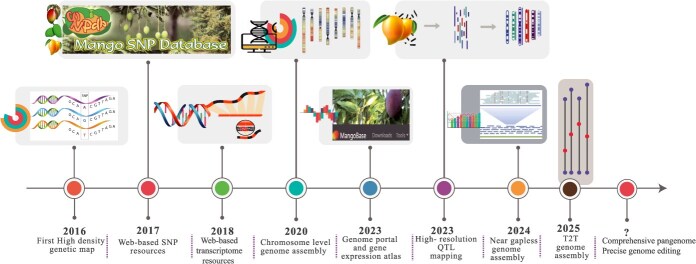
Important developments in mango genomics and future priorities. (Mango SNP Database and MangoBase images are downloaded from relevant websites).

Mango has been genomically understudied and lacks a detailed understanding of the genetic basis of complex agronomic traits. However, significant progress has been made in the last decade in reference genome assembly [2, 19–24], genetic maps with all 20 linkage groups [25], transcriptomes [26–32], molecular analyses of germplasm collections [30, 33, 34], gene function [35] structural variation identification [36], and quantitative trait loci (QTL) mapping to identify loci underlying key traits [37] (Fig. 1). Recent advancements in genomics, machine learning, and genome editing have led to new concepts and discoveries in crop domestication and breeding. The integration of these innovations will enhance the efficiency of breeding [38]. Here, we summarize the progress made in mango breeding by genetic mapping, genomics, genetics, and functional genomics and a way forward for future breeding programs.

## The origin of the genus *Mangifera*

The genus *Mangifera* belongs to the Anacardiaceae family, which has 73 genera and 850 species. Mostly of tropical origin; however, some have temperate origins. The Malay Peninsula (South of the Kangar Pattani line to the Bismarck archipelago east of New Guinea) contains the highest number of species of the Anacardiaceae family [39]. The Anacardiaceae family diverged from its sister family, Burseraceae, during the late Cretaceous (~100–66 million years ago) due to continental vicariance, with their most recent common ancestor likely distributed across the Northern Hemisphere. Both families share similar stem age, but the speed of diversification differs significantly—Anacardiaceae shows a broader range of fruit morphologies and dispersal strategies, and has species that can resist freezing temperatures. This endorses that Anacardiaceae adapted more frequently to shifting climates and niches, likely due to greater intrinsic evolutionary lability or exposure to heterogeneous selective pressures [40].

Within Anacardiaceae, the genus *Mangifera* stands out for its ecological and economic significance. Based on the existing diversity in Borneo, the Malay Peninsula, and Sumatra, this region is considered the origin of the genus *Mangifera* [41], where it is believed to have emerged during the Eocene or possibly the Cretaceous period. The most recent classification of *Mangifera* is based on floral morphology. Accordingly, genus *Mangifera* has two subgenera—*Mangifera* and *Limus* (Marchand) Kosterm. The evolutionary origins of the genus *Mangifera* and its species remain uncertain, and even cladistic analyses cannot resolve their precise ancestry. It is surmised that two subgenera may have different origins. Out of a total of 69 species, 47 are included in subgenus *Mangifera*, 11 in *Limus* (Marchand), and the position of 11 species is still uncertain. Subgenus *Mangifera* is subdivided into four sections—*Marchandora Pierre*, *Euantherae Pierre*, *Rawa Kosterm*, and *Mangifera Ding Hou*. These sections contain 1, 3, 9, and 34 species, respectively [6, 41]. Further details on classification, conservation status, and species with important breeding traits are provided in Table 1.

**Table 1:** Taxonomy of genus *Mangifera* [6]: edibility, conservation status, and breeding traits.

**Subgenus**	**Section**	**Species**
*Mangifera*	*Marchandora* Pierre	***M. gedede* Miq** (1)
	*Euantherae* Pierre	*M. calneuron* Kurz, ***M. cochinchinensis* Engler**, ***M. pentandra* Hooker f.** (2)
	*Rawa* Kosterm	***M. andamanica* King,** *M. gracilipes*, ***M. griffithii* Hooker f*.*,** M. merrillii Mukherji*, ***M. microphylla* Griff. ex Hooker f.**, *M. minutifolia* Evard*., *M. nicobarica* Kosterm*., *M. paludosa* Kosterm*., *M. parvifolia* Boerl. & Koorders
	*Mangifera* Ding Hou	***M. altissima* Blanco** (3), *M. applanta* Kosterm*.*, *M. austro-indica* Kosterm*., *M. austro-yunnanensis* Hu, *M. casturi* Kosterm* (4), *M. collina* Kosterm*., *M. dewildei* Kosterm*., *M. dongnaienis* Pierre*, *M. flava* Evard*.*, ***M. indica* L*.*,** *M. lalijwa* Kosterm*., ***M. laurina* Bl***.* (5), *M. linearifolia* (Mukherji) Kosterm*.*, *M. longipetiolata* King, *M. magnifica* Kochummen, *M. minor* Bl., ***M. monandra* Merr***.* (6), *M. mucronulata* Bl*.*, *M. oblongifolia* Hooker f*., M. orophila* Kosterm*.*, *M. pedicellata* Kosterm*.*, *M. pseudo-indica* Kosterm*.*, ***M. quadrifida* Jack, *M. rigida* Bl***.*, *M. rubropetala* Kosterm*.*, ***M. rufocostata* Kosterm***.* (7), ***M. similis* Bl***.* (8), *M. sulawesiana* Kosterm*.* **, *M. sumbaewanensis* Kosterm*., ***M. sylvatica* Roxb***.*, ***M. swintonioides* Kosterm***.* (7), *M. timorensis* Bl., *M. torquenda* Kosterm*., ***M. zeylanica* (Bl.) Hooker f*.***
*Limus* (Marchand) Kosterm.		*M. blomesteinii* Kosterm*.*, ***M. caesia* Jack** (9), ***M. decandra* Ding Hou** (1), ***M. foetida* Lour*.*** (10), ***M. kemanga* Bl*.*, *M. lagenifera* Griff*.***, *M. leschenaultii* Marchand, ***M. macrocarpa* Bl*.***, ***M. odorata* Griff*.*** (11), ***M. pajang* Kosterm*.*** (12), *M. superba* Hooker f*.*
Uncertain		*M. acutigemma* Kosterm*.*, *M. bompardii* Kosterm*.*, *M. bullata* Kosterm*.*, *M. campospermoides* Kosterm*, *M. hiemalis* Liang Jian Ying, *M. maingayi* Hooker f*.*, ***M. persiciformis* Wu & Ming**, *M. subsessifolia* Kosterm*.*, *M. taipu Buch*.-Hamilton, *M. transversalis* Kosterm., *M. utana* Utana
**Description of special traits and symbol legend** (1) Tolerant to waterlogged conditions and can be used as rootstock. *M. reba* Pierre, *M. camptosperma* Pierre, and *M. inocarpoides* Merr. and Perry are its synonyms. (2) High and consistent fruit yield due to the prevalence of hermaphrodite flowers. (3) Insect tolerant (Seed borers and hoppers). (4) Heavy and regular bearer with small, black, and sweet fruit. (5) Also known as *M. longipes*, it is immune to anthracnose. (6) Unripe fruit can be eaten (7) Off-season bearing (8) Stone-free fruits (9) The pulp is soft, milky-white, sweet, and fragrant. (10) Less fiber and sweet taste (11) Distinct aroma, firm texture, sweet–sour taste, and can grow in high-rainfall and humid conditions (12) Fruit peel can be easily removed, like a Banana. Species names in bold denote edible species * Endangered species ** Extinct in the wild		

Sources [6, 41, 46, 47].

**Figure 2:**
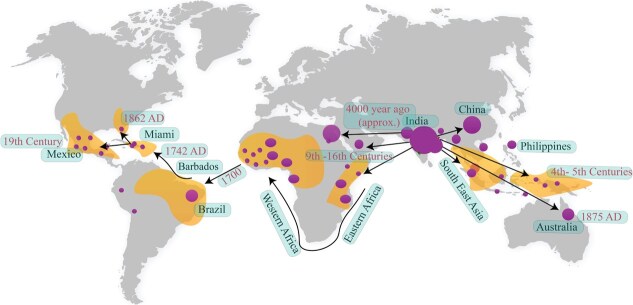
A holistic exploration of the origins and global dispersal patterns of mangoes. Yellow shapes represent regions including multiple countries, while the size of the purple circles indicates germplasm diversity. Adapted and expanded from concepts previously discussed in [54].

Mango was grown in Indo-Burmese and Southeast Asian regions for an estimated 4000 years and later spread to other parts of the world (Fig. 2) [34] such as America and Africa. The cultivated mango (*M. indica* L.) is thought to have come from Eastern India, particularly in Assam, to Burma or the Malayan region. Mango may have naturally originated in India as a wild fruit [42]. Indo-Burma was also considered the center of origin [43]. The historical records, 60-million-year-old fossil remains of carbonized mango leaves [44], distribution of related species and genera, and the existence of multiple varieties and wild species strongly endorse that Indian subcontinent is the geographical origin of *Mangifera*. The fossil remains of leaf imprints established that Assam was the origin of *M. pentandra* [45]. Some studies report the origin of wild common mango trees in Andaman and Nicobar Islands, Bangladesh, Myanmar, and Northeastern India. Many semiwild mango germplasm resources are found in the forests of the Indian subcontinent. Northeast India, Borneo, Peninsular Malaya, and Sumatra have the maximum species diversity of the *Mangra* genus. The forests of Borneo, Thailand, the Malay Peninsula, Indo-China, the Indonesian archipelago, and the Philippines house seedling variations with primitive traits, i.e. dwarf growth habit and polyembryony, along with a rich array of species.

The genus *Mangifera* evolved over a wide geographic range, with the Malay Peninsula considered as the origin’s hub and Siam, Burma, Indo-China, and Southeast Asia as the region with the greatest diversity [8, 48]. The Malay Peninsula, Sunda Islands, and Eastern Peninsula have reported 19, 16, and 14 *Mangifera* species, respectively, indicating that these regions have the maximum diversity. The noted species include *M. indica* and its close relatives like *M. sylvatica*, *M. odorata*, *M. pentandra*, *M. foetida*, *M. caesia*, and *M. caloneura*. The region between Indo-China and Myanmar is one of the major centers of diversification, due to the presence of many Euantherae (*M. cochinchinensis*, *M. caloneura*, and *M. pentandra*) section species [49].

Multiple genomic tests confirm that *M. indica* exhibits exceptional genetic diversity compared to many horticultural crops, particularly among Southeast Asian cultivars genetically divergent from other *M. indica* germplasms. Phylogenetic studies have yet to decipher the relationship between *M. indica* and its closely related species, but studies like [34] have made significant progress in understanding global mango diversity and domestication history. According to the maximum-likelihood topology, it is suggested that mango cultivars from Southeast Asia diverged earlier than those from other regions worldwide. Previous hypotheses include independent domestication in India and Indochina and initial cultivation in Southeast Asia, with subsequent improvements in India. In a recent study, multiple population genetic analyses were conducted on 208 accessions of *M. indica* [50]. The results revealed the presence of several genetic clusters, with one particular cluster showing significant genetic differentiation. Notably, this genetically differentiated cluster comprised accessions that had been imported from the Southeast Asia region. There are controversies about the center of origin of cultivated mango, some argue for India as a single origin [51, 52] while others proposed Southeast Asia [1, 41]. However, based on recent studies [19, 34, 53] it is commonly accepted that mango has two centers of origin: India and Southeast Asia (Fig. 2).

Apart from the two main centers of origin, mango diversity can also be observed in other parts of the world, such as Africa, Australia, and the USA. After the first mango arrived on Florida’s shores in 1833, it emerged as a major secondary center for mango production, in the late 19th and early 20th centuries. Monoembryonic Indian cultivar (Malgoa) showed a polyembryonic nature in the USA [11]. Early seedling selections from ‘Malgoa’ were designated ‘Haden’, and later on world-leading monoembryonic cultivars ‘Keitt’,‘Kent’, and ‘Tommy Atkins’ were developed from this [55]. Numerous new Floridian cultivars were introduced and promoted to meet the taste preferences of Western consumers. These cultivars were selectively bred for their appealing red blush coloration, mild flavor, and high fruit weight. Despite the popularity of these cultivars, there remains a demand for further improvements in taste and quality. As such, breeding programs in Australia, South Africa, Israel, Brazil, and China are actively engaged in developing new and improved mango cultivars. These programs are dedicated to creating mango varieties that are even more delicious, visually appealing, and adaptable to a wider range of growing conditions [56]. Table 2 summarizes the phenotypic diversity of leading mango cultivars, showing variations in fruit morphology (size, shape, skin, pulp color), fruit quality (pulp texture, sweetness, aroma, fiber content), shelf life, and yield potential across major mango-growing regions. This global diversity displays the crop’s adaptability and value for targeted breeding.

**Table 2:** Global overview of phenotypic diversity in mango cultivars.

**Trait**	**Phenotypic diversity**	**Cultivar(s)**	**Country/Region**
**Fruit size**	Small (100–300 g)	Ataulfo Zafiro, Ataulfo Diamante, Alphonso, Dashehari, GuiQI Mango, Yuexi NO.1	Mexico, India, China
	Medium (300–450 g)	Angie, Mallika, Nam Doc Mai, Calypso, Orli, Langra, Guifei, Nan Klang wun	USA, India, Thailand, Israel, Pakistan, China,
	Large (>450 g)	Noa, Keitt, Kent, Omer, Tommy Atkins, Sindhri, Chenab Gold, JinHuang mango, Longjing mango, Hongxiangya	USA, Israel, Pakistan, China
**Fruit shape**	Oblong	Ataulfo, Duncan, Harders, Tota Pari, JinHuang mango, Hongxiangya, Chenab Gold	Mexico, USA, India, China, Pakistan
	Elliptic	Kensington Pride, Amrapalli, Zihua	Australia, India, China
	Roundish	Angie, Orli, Agam, R2E2, Renong NO.1, Keitt	USA, Australia, Israel, China
	Ovoid	Kent, Cogshall, Chaunsa, Langra, GuiQI Mango, Tainong NO.1, Siji mango, Sensation, Carabao	USA, India, China, Pakistan, Thailand
	Obovoid	Nam Doc Mai, Edward, Alphonso, Sannian Mango	Thailand, USA, India, Philippines
**Pulp texture**	Soft	Ataulfo, Angie, Langra, Dashehari, GuiQI mango, Hongyu, Nan Klang wun, Chenab Gold	Mexico, India, China, Thailand, Pakistan
	Intermediate	Calypso, Kent, Nam Doc Mai, Alphonso, JinHuang mango, Guifei, Dashehari, Sufaid Chaunsa	Australia, USA, Thailand, China, India, Pakistan
	Firm	Keitt, Duncan, Gouveia, Tommy Atkins, Haden, Tainong NO.1, Renong NO.1	USA, Brazil, India, China
**Skin color (ripe)**	Green	Kensington Pride, Langra, GuiQi Mango, Hongyu, Rad	Australia, Pakistan, China, Thailand
	Yellow	Ataulfo Zafiro, Nam Doc Mai, Duncan, Mallika, Sindhri, Chenab Gold	Mexico, Thailand, India, Pakistan
	Greenish-yellow	Totapari, JinHuang mango, Siroli	India, China, Pakistan
	Green with red blush	Tommy Atkins	USA
	Green with purple patches	Cavallini	Israel, Australia
	Dark red	Ah Ping, Agam, Sensation, Irwin, Tommy Atkins	USA, Israel
**Pulp color**	Light yellow	Favo de Mel, Nang Klang Wan, Baiyu mango	Brazil, Thailand, China
	Golden yellow	Kent, Keitt, Ataulfo Diamante, Ataulfo Zafiro, Edward, Kensington, Carabao	USA, Mexico, Australia, Philippines
	Yellow orange	Kent, Mallika, Palmer, Edward, Guifei, Hongyu	USA, India, China
	Orange	Madame Francis, Ah Ping, Duncan, Mallika	Canary Islands, USA, India
	Yellow	Tommy Atkins, Ataulfo, JinHuang mango, Sannian mango	USA, Mexico, China,
	Light orange	Kensington Pride, Maha Chinook	Australia, Thailand
	Dark orange	Tali, Mcheso, Dashehari	Israel, Myanmar, China
**TSS (°Brix)**	Low (10–14.0)	Ataulfo Zafiro, Edward, Renong NO.1, Hongyu	Mexico, USA, China
	Medium (14.1–18.0)	Nam Doc Mai, Kent, Totapari, Guifei	Thailand, USA, India, China
	High (18.1–22.0)	Alphonso, Chaunsa, Angie, Mallika, Favo de Mel, JinHuang mango, Tainong NO.1	India, Pakistan, USA, Brazil, China,
	Very high (>22.0)	Papo de Peru 2, GuiQi Mango, Azeem Chaunsa	Brazil, China, Pakistan
**Aroma**	Mild	Kent, Keitt, Omer, Maha Chinook, Renong NO.1, Nan Klang wun	USA, Israel, China
	Intermediate	Calypso, Cogshall, Nam Doc Mai, JinHuang Mango, Guifei Hongyu	Australia, USA, China, Thailand
	Strong	Alphonso, Kensington Pride, Mallika, GuiQi Mango, Repin NO.16, Tainong NO.1, Sannian mango	India, Australia, China
**Shelf life**	Short (<7 days)	Alphonso, Carabao, GuiQi Mango, Guifei	India, Philippines, China
	Medium (7–14 days)	Nam Doc Mai, Angie, Ataulfo, Sannian mango, Keitt, Alphonso	Thailand, USA, Mexico, India
	Long (>15 days)	Keitt, Tommy Atkins, Kent, JinHuang mango, Tainong NO.1, Renong NO.1, Chenab Gold	USA, China, Pakistan
**Yield potential**	High	Tommy Atkins, Keitt, Calypso, Sindhri, GuiQi mango, JinHuang mango, Nan Klang wun	USA, Australia, Pakistan, China, Thailand
	Moderate	Mallika, Nam Doc Mai, Kensington Pride, Hongyu, Sannian mango	India, Thailand, Australia, China
**Fiber quantity**	Absent	Ataulfo, Alphonso, Nan Klang wun, Nam Doc Mai	Mexico, India, Thailand
	Low	Kensington Pride, Maha Chinook, Keitt, Mallika, Chaunsa, Dasheri, JinHuang mango	USA, Australia, India, Pakistan, China
	Intermediate	Cogshall, Kent, ‘Papo de Peru 2, Madame Francis, GuiQi mango, Tainong NO.1	USA, Brazil, Canary Islands, China
	High	Totapuri, Sannian mango, Dongpo mango	India, China

Sources [57–63]

## Domestication

The domestication history of fruit crops has played a vital role in shaping their characteristics and availability [64]. Central to this process is the concept of domestication syndrome—a suite of heritable traits that distinguish cultivated plants from their wild progenitors, arising from human selection [65]. Historical records suggest that mango trees initially have small, thin-fleshed fruits, while centuries of human selection led to larger fruits with more edible pulp [49]. These morphological changes mark some of the earliest domestication traits. Modern breeding programs have expanded the domestication syndrome to include traits such as reduced tree vigor, regular and high yields, disease resistance, improved transportability, and extended shelf life, aligning selection goals with both production efficiency and consumer preferences [14, 66]. However, studies into the primary bottleneck related to mango’s initial domestication are hindered by the inaccessibility of wild *M. indica* populations, and the history of human-mediated migration into new areas allows for investigating whether secondary genetic bottlenecks occurred during successive founder events. Although many studies have shed light on the molecular diversity and genetic structure of mango cultivars within specific regions, including Brazil [67], China [68], Kenya [69], Myanmar [70], Colombia [71], Iran [72], and particularly India [73–75], only a few studies have investigated mango cultivars across a wide geographical range.

Studies performed in the 2000s based on the microsatellite genetic markers found Southeast Asian mango cultivars were more differentiated from other populations [18, 30]. Some studies revealed that the domestication history of mango is more complex than previously thought. It proposes two trends observed in perennial fruit crop domestication: multiple domestications and hybridization with related species. These trends are common in perennial crops and occur over a broader geographical scale and a longer period compared to annual species. This indicates the presence of two cultivated gene pools in mango, contributing to regions of increased diversity outside the presumed centers of origin. Furthermore, it suggests that not all the genetic variation present in current mangoes can be traced back to India [34].

Over the past decade, numerous national and international initiatives have been dedicated to large-scale genotyping of plant genetic resources. Table 3 presents a compilation of studies conducted in different countries, aiming to investigate the genetic resources available in those regions or comparison with genetic resources available in other countries. Overall, simple sequence repeats (SSRs) are marker types widely used for examining levels of diversity and polymorphism [76]. The single nucleotide polymorphism (SNP) genotyping arrays are very helpful for pedigree identification and have been developed in many horticultural crops, such as cherry, peach, grape, avocado, apple, potato, rose, walnut, pear, and strawberry [77–80]. SNP arrays have not been specifically designed for parentage analysis and have been limited to a few SSR markers, and due to this many popular open-pollinated mango cultivars either have a single known parent or lack parentage information entirely. In recent years, the availability of diverse genomic resources, SNP genotyping arrays, and cost reductions in sequencing have made the genotyping of open-pollinated seedlings more attractive for mango breeding programs.

**Table 3:** Details of studies performed using various techniques for genomic diversity analysis in different parts of the world.

**Country**	**Technique**	**Total mango Cvs and their origin**	**Conclusion**	**References**
**Australia**	SSR markers	254 accessions of *M. indica* L. and related *Mangifera* species from 12 diverse geographic areas	133 alleles were detected using 11 SSR markers. The accessions were divided into four major nodes, indicating geographical origins. Twenty-four unique genotypes were recognized for 50 trees previously assigned different accession names. No relationship was detected between SSR markers and embryony.	[30]
**Bangladesh**	Multivariate analysis	23 local genotypes	The analysis divided the genotypes into five clusters, with 15 confined to just two.	[81]
**Brazil**	RAPDS	35 accessions (Brazil), 6 (USA), 1 (India)	The unweighted pair group method utilizing arithmetic average cluster analysis identified 55 polymorphic loci and suggested five genotypic categories.	[82]
**China**	SCoT markers	168 mango accessions (52 Cvs introduced from different countries, i.e. USA, Australia, and 116 indigenous accessions present at Guangxi University)	45 primers generated 337 fragments, and 244 (72.4%) exhibited polymorphism. The dendrogram divided accessions into two clusters with 89% of genetic diversity occurring within populations versus 11% among populations.	[83]
**China**	WGS	224 mango accessions (wild and cultivars) from 22 countries	*M. himalis* J.Y. Liang was reclassified into *M. indica*. Population structure and differentiation analysis divided Chinese accessions into two distinct pools.	[53]
**Egypt**	SSR and EST-SSR	28 mango Cvs (4 native Egyptian CV & 24 being grown in Egypt)	Genetic polymorphism and morphological traits both showed significant variation, ranging from 0.71% to 100%.	[84]
**India**	Multiple markers (65 RAPD, 25 ISSR, 23 DAMD, 22 SCoT, 33 CBDP, and 40 SSR)	70 selected Indian mango genotypes based on geographic and fruit status.	The highest genetic diversity was seen in the East Indian populations. The indigenous genotypes showed more potential for exploiting the unique and favorable alleles. The maximum genetic variability was detected with SSRs.	[76]
**Indonesia**	Morphology and RAPD markers	82 native Cvs	Morphological studies divided all Cvs into three main groups, i.e. Kidang Kencono, Kopek, and Carang. RAPD suggested a 2%–31% range of genetic variability, and 9 clusters having 75% similarity were formed.	[85]
**Japan**	SSR markers	120 mango genetic resources available in the country (imported from 17 different countries)	46 polymorphic SSR markers were used, and 10 sets of three SSR markers distinguished 83 genotypes, excluding synonymous and identical accessions. The analysis revealed three clusters, showing genetic differentiation between Indian and Southeast Asian varieties, with Floridian varieties being closer to the Indian variety.	[10]
**Mexico**	AFLP	41 local mango Cvs	High levels (>84%) of polymorphisms were detected among accessions. Based on AMOVA analysis, significant genetic differences were detected among and within mango accessions. Genetic differences were found to be related to their geographical origins.	[86]
**Nigeria**	SSR markers	17 selected mango Cvs present in Nigeria	The seven polymorphic primers identified a total of 21 alleles, with each locus containing 2–5 alleles and an average of 3.0 alleles per locus.	[63]
**Pakistan**	SSR Markers	15 Indigenous Cvs	Utilizing 12 SSR primers, the accessions showed coefficients of similarity ranging from 75% to 100%, indicating inbreeding and a few parents.	[87]
**Thailand**	SSR anchored primers	24 mango Cvs (13, 4, 3, 1, and Thai, Florida, Indian, Indonesian, and Philippine, respectively)	40 primers were used, and seven primers produced polymorphic DNA amplification patterns. Two Thai Cvs (Nong Saeng and Nang Klangwan) showed more genetic diversity than other Cvs, and based on the dendrogram, 11 closely related Thai Cvs were divided into three groups.	[88]
**USA, Senegal, Thailand, Australia**	Simplified visual analysis plus SNP markers	Different *Mangifera* species, accessions from half-sibling populations, and 2232 accessions	272 SNP markers identified >520 000 Genotypes. The affinity propagation method divided genotypes into 258 groups. A simple visual method suggested that no more than 30 SNPs are required for the accurate identification of a CV.	[33]
**USA**	Microsatellite markers	208 accesions of mango (203, *M. indica*: 2, *M. griffi thii* Hook f.: and 3, *M. odorata* Griff.)	For the *M. indica* population, 25 microsatellite loci displayed an average of 6.96 alleles per locus and an average polymorphism information content score of 0.552. Estimates put the collection’s overall propagation error at 6.13%. The Florida Cvs were more closely related to Southeast Asian Cvs than to those from India.	[18]
**Vietnam**	RAPD and ISSR markers	10 mango samples from different regions	Using 10 ISSR and 10 RAPD markers high polymorphic levels were detected; ISSR (98.1%) and RAPD (91.9%), and 145 and 162 loci were generated, respectively.	[89]

**Abbreviations:** Cvs (Cultivars), SSRs (Simple Sequence Repeats), RAPDS (Random Amplified Polymorphic DNA), SCoT (Start Codon Targeted), EST-SSR (Expressed Sequence Tag-Simple Sequence Repeat), DAMD (Directed Amplification of Minisatellite-region DNA), ISSR (Inter Simple Sequence Repeat), CBDP (CAAT box-derived polymorphism) AFLP (Amplified Fragment Length Polymorphism), SNP (Single Nucleotide Polymorphism), WGS (Whole-Genome Resequencing)

**Table 4:** Comparative statistics of available mango genomes.

**Mango Cv.**	Amrapali	Hong Xiang Ya	Alphonso	Irwin	Tommy Atkins	Irwin Hap1	Irwin Hap2	Irwin collapsed	Alphonso ALT2T_HAP1	Alphonso ALT2T_HAP2
**Sequencing technology**	PacBio, Illumina, BioNano	PacBio CLR+ Illumina	PacBio Sequel II + HiSeq2000 and MiSeq + HiC	PacBio CLR + Illumina + Hi-C	Illumina HiSeq	PacBio HiFi	PacBio, HiFi	PacBio HiFi	PacBio HiFi HiC, ONT	PacBio HiFi HiC, ONT
**Assembly method**	FALCON (v0.2.2)	Falcon version (v0.3.0)	Canu (v1.8)	MECAT (v1.2); Polish (v1.22)	DeNovo Magic (v3.0)	HiFiasm (v1)	HiFiasm (v1)	HiFiasm (v1)	HiFiasm (v0.16), Juicer (v1.5), 3D-DNA (v180922)	HiFiasm (v0.16), Juicer (v1.5), 3D-DNA (v180922)
**Genome coverage (x)**	289.6	388	240	61.96	180	204	204	204	Hifi:88.4 ONT:149.4	Hifi:88.12 ONT:148.88
**Genome size (including unplaced scaffolds) (Mb)**	411.3	371.62	392	396	374.8					
**Assembly size (Mb)**	290	367	357	375	328	354	354	365	346.22	347.59
**Assembly level**	Chromosome	Chromosome	Chromosome	Chromosome	Chromosome	Chromosome	Chromosome	Chromosome	Chromosome	Chromosome
**Number of contigs**	3714	120	420	3022	17 187	4711	1515	4642	397	70
**Contig N50 (Mb)**	0.955	4.82	3.50	1.03	0.040	13.21	15.45	14.98	17.03	17.96
**Number of scaffolds**	2314		252	1305	2565	23	23	23	390	65
**N50 scaffold (Mb)**	12.1	18.78	17.6	1.03	16.2				18.06	19.25
**Telomers**						37	37	40	37	36
**BUSCO% (Embryophyta__odb10)**	97.4	93.3	95.9		94.6	98.1	99	99.2	99.1	99.1
**No. of predicted genes**	46 395	34 529	41 251	36 756	26 616	34 659	33 230	35 220	41 423	40 794
**Accession number**	PRJNA300605	PRJCA002248	PRJNA487154	CRA004336	PRJCA005296	GWHESFJ00000000	GWHESFK00000000	GWHEQCT00000000	PRJNA1218505, PRJCA035721	PRJNA1218506, PRJCA035722
**Public source**	NCBI	NCBI	NCBI	NGDC	NCBI	NGDC	NGDC	NGDC	NCBI, NGDC	NCBI, NGDC
**Reference**	94	22	19	20	2	21	21	21	23	23

**Abbreviations:** BUSCO (Benchmarking Universal Single-Copy Orthologs), HiFiasm (Highly Efficient Assembler for Long-Reads Sequencing Data), ONT (Oxford Nanopore Technologies), NCBI (National Center for Biotechnology Information), NGDC (National Genomics Data Center)

## Progress and advances in genomics-based methods

### Mango genomic databases

Over the last decade, many mango genomes have been sequenced and several SSR and SNP markers have been produced. Mango genomic databases have been created to effectively store this growing genomic data. In 2017, the first SNP database for mango, known as the *M. indica* SNP database (MiSNPDb; http://webtom.cabgrid.res.in/mangosnps/), was developed [90]. This database provides information about 1.25 million SNPs discovered from 84 distinct mango cultivars using ddRAD-Seq approach. In 2018, Qamar-ul-Islam and his team developed a cutting-edge Mango Genomic database called MGdb after a comparative transcriptome analysis of four mango cultivars, namely ‘Langra’ (Pakistan), ‘Shelly’ (Israel), ‘Zill’ (China), and ‘Kent’ (Mexico). The database includes transcriptomic resources (e.g. 30 000–85 000 unigenes) that [91], while limited compared to modern telomere-to-telomere (T2T) assemblies, offer foundational insights into mango’s genetic diversity. In 2023, a comprehensive resource for mango genomics called MangoBase (https://mangobase.org/) was established [92]. This platform is solely dedicated to mango genetics and provides an array of interactive bioinformatics tools, sequences, and annotations to analyze, visualize, and retrieve omics data. One of its notable features is a gene expression atlas that comprises 12 datasets and 80 experiments analyzed based on the ‘Tommy Atkins genome’ reference, which is linked to their gene references and annotations. Overall, these powerful tools will help researchers gain a deeper understanding of the genetic makeup of mangoes and explore new possibilities for developing improved cultivars with desirable traits.

## Genome assemblies and comparative genomics

Plant genomes provide evidence of evolutionary history, whole-genome duplication, population processes, and other factors [93]. Early attempts to assemble the mango genome were hindered by its high heterozygosity (~2.5%) [94]. However, current sequencing and assembling technologies are capable of assembling highly heterozygous genomes into two separate haplotypes. The first attempt to assemble the mango cultivar ‘Amrapali’ genome was made using Illumina MiSeq overlapping paired-end reads (100–150 bp). However, the assembly size of 492 Mb in 211 141 contigs exceeded the estimated size of the mango genome (439 Mb), suggesting redundancies in the assembled contigs [95]. Later, the raised issues were resolved by utilizing PacBio single-molecule real-time (SMRT) long-sequence reads for genome assembly and allowed for long overlaps of 500 bp and a high mismatch rate of 15% to accommodate the heterozygosity [54]. This approach resulted in the first draft genome assembly of 323 Mb for the mango cultivar ‘Amrapali’ (NCBI: LMWC00000000 v1). Later in the same year, the draft genome assembly (407 Mb) of the prominent Australian mango cultivar ‘Kensington Pride’ was developed based on Illumina short-read sequencing [96], the issue of heterozygosity was not addressed, and contig redundancy remained a concern.

Due to the recent advancements in sequencing techniques and bioinformatics tools, significant advancements have been made in the complete genome sequencing of mango cultivars. The complete genome sequencing of the ‘Hong Xiang Ya’ cultivar resulted in a high-quality genome assembly, comprising 371.6 Mb of genomic sequence (Table 4). Utilizing the available genetic map, the genome was assembled onto its respective chromosomes, achieving a remarkable anchoring of ~98.77% of the genome assembly onto 20 pseudo-chromosomes [22]. Furthermore, a better chromosome-scale genome assembly of the mango cultivar ‘Alphonso’ was successfully generated and is being used as a reference genome [19]. Moreover, the 10× genomics long-read sequencing was used for generating a high-quality genome assembly of ‘Tommy Atkins’. The assembly included ~86% of the ~439 Mb haploid mango genome. Approximately 3.3 M SNPs were identified using the mapping population of ‘Tommy Atkins’ and ‘Kensington Pride’, and 28 potential genes associated with fruit size were identified [2]. Wijesundara *et al*. [21] generated a good quality, near-gapless chromosome-level (365 Mb) assembly of ‘Irwin’ with only high coverage (204×) HiFi. Recently, a gapless, chromosome-level genome assembly (377.6 Mb) of the mango landrace ‘San Nian Mang’, consisting of a single contig per chromosome, was reported [24]. In addition, a haplotype-resolved, T2T genome assembly of the cultivar ‘Alphonso’ has been generated using a combination of PacBio HiFi, Oxford Nanopore (ONT), and Hi-C sequencing data [23], providing the high-quality mango reference genome to date.

Due to the wide genetic diversity in *M. indica*, single- or multiple-reference genomes may not be enough for the in-depth genetic diversity analysis of the species; for this, a pangenome is required. A pangenome refers to a detailed collection of genomes from a species, achieved by comparing numerous genomes. The construction of a pangenome facilitates the determination of various essential aspects, such as the size of a core genome (the conserved portion among related genomes), the overall size of the pangenome, and the extent and characteristics of variations within a species or genus [97]. This in-depth understanding contributes to advancements in our knowledge of species/genus evolution and aids in studying agronomic traits. Recently, a draft pangenome of mango was constructed using three cultivars [98] but not of high quality. Identification of disease-resistance alleles in mango cultivars and their wild relatives is important for improving mango crop quality. This can be achieved by constructing a pangenome using wild and cultivated accessions. In the future, more T2T genomes and a comprehensive pangenome of the genus *Mangifera* are needed for more insights into mango genomics and breeding.

With the accessibility of complete genome sequencing data, opportunities for comparative genome analysis are opening up to uncover gene networks, transposable elements, intron–exon boundaries, novel biological processes, molecular markers associated with economically significant traits for breeding purposes, and structural variants [36, 99–102]. Cortaga *et al*. [36] sequenced the whole genomes of three mango species native to the Philippines (Carabao, Huani, and Paho). Genome-wide variants were identified by mapping 93%–95% of the quality-filtered reads with sequence coverage of 2.91× to 4.30× to the Alphonso and Tommy Atkins mango genomes. They identified >2.3 million SNPs, >199 000 insertions, and > 199 000 deletions in each species. To identify genomic and structural variants, few studies have been performed, and more studies are thus needed in the future.

Besides, the mitochondrial and chloroplast genomes also provide valuable information for understanding the evolutionary history of fruit plants [102, 103]. Recently, phylogenetic relationships within the genus *Mangifera* were determined using whole chloroplast and nuclear genome sequences [104]. Thirteen samples representing 11 *Mangifera* species were sequenced, revealing that the chloroplast genomes of *M. altissima*, *M. caloneura*, *M. applanata*, and *M. lalijiwa* were nearly identical to that of *M. indica* (99.9% sequence similarity). The close genetic relationship indicates a common ancestry and potential cross-hybridization between *M.* wild relatives and *M. indica*.

## Genetic basis of complex agronomic traits

Plant breeding traditionally relies on phenotypic selection but can be time-consuming, especially when dealing with fruit trees. The most time-consuming phase in this process is the transition from the juvenile stage to a fruit-bearing tree, which typically takes several years in mango trees [ 8, 105, 106]. By using molecular genomic tools, there is an opportunity to assess the genetic diversity of prospective parent plants, identify markers linked to significant horticultural traits, and enhance the speed of mango breeding programs [66].

Early selection and use of molecular markers through marker-assisted selection (MAS) can significantly streamline the breeding process. A high-density genetic map is required for QTL mapping. Kashkush *et al*. [107] made an initial attempt to generate a genetic map of mangoes. Subsequently, attempts were made by other scientists [108, 109] for improvements, and a high-resolution genetic map with the potential for future use was generated in 2016 [110]. A comparison of the average distance between this map and others demonstrated the possibility of constructing a high-density genetic map capable of fine QTL mapping and MAS in mango. Later on, Kuhn *et al*. [66] developed a high-density genetic map for mango, allowing for the exploration of significant associations between traits and SNP markers (Table 5).

**Table 5:** Details of available genetic maps in mango.

**Genotyping technology**	**Population size**	**Number of markers**	**Total genetic distance (cM)**	**Distance between markers (cM)**	**Number of LGs**	**Linked QTLs**	**References**
**RFLP and AFLP**	31	847	1437.7	10.4			[108]
**AFLP**	29	34	NA	4.75			[107]
**AFLP**	60	81	354.1	4.37			[109]
**Microsatellite and RFLP**	31	76	529.9	6.97			[111]
**SLAF-seq**	173	6594	3148.28	0.48	20		[110]
**SNP markers**	775	726	2890.6	4.13	20	Polyembryony, branch habit, bloom, ground skin color, blush intensity, beak shape, pulp color	[66]
**SNP Markers**	94	4361	2982.75	0.68	20	Fruit color and firmness	[112]

**Abbreviations:** cM (centiMorgan—unit of genetic linkage), RFLP (Restriction Fragment Length Polymorphism), AFLP (Amplified Fragment Length Polymorphism), SLAF-seq (Specific-Locus Amplified Fragment Sequencing), SNP (Single Nucleotide Polymorphism) LGs (Linkage Groups), QTLs (Quantitative Trait Loci)

Some studies have highlighted the important role of genetic structure in trait mapping accuracy and breeding efficiency. Wilkinson *et al*. [50] identified four distinct genetic clusters in the Australian mango gene pool, with the most differentiated group comprising Southeast Asian accessions. This population structure was strongly linked with key traits like trunk circumference and fruit blush color, demonstrating how shared ancestry can create spurious genotype–phenotype linkages. For accurate QTL mapping and genomic selection, it is therefore essential to account for population structure to avoid false marker–trait associations. These findings underscore that breeding programs must characterize genetic backgrounds (e.g. the unique Southeast Asian gene pool) to improve predictive accuracy for complex traits like fruit quality. Fruit size, firmness, aroma, and color are important fruit quality traits that need to be examined at the genomic level for cultivar improvement [2]. Fruit quality-related traits are quantitative traits and are influenced by multiple genes. Transcriptomic studies [113, 114] have identified some genes potentially involved in fruit color, flavor, ripening duration, and storage period. However, the lack of functional validations suggests that the genetic basis of these traits is largely unknown. Recently, Srivastav *et al*. [112] constructed two high-density linkage maps based on segregating female and male parents and performed high-resolution QTL mapping. They reported that QTLs for fruit peel color are located at Chr 3 (99.63 cM) and 18, and pulp firmness on Chr 11 (94.82–95.19 cM) and 20.

Flowering at a favorable time gives a desirable crop yield, while the flowering mechanism in mango is very complex and challenging for growers, physiologists, and breeders [115]. Like many other perennial fruit crops, mango tends to be alternate (Heavy fruit in one year (ON) and less fruit (OFF)) or irregular bearing. This phenomenon poses severe losses to farmers and is also undesirable from a breeding point of view. Alternate bearing is controlled by environmental, genetic, physiological, and biochemical factors [116]. In mango exploring the genetic basis of alternate bearing is difficult due to some inherent factors influencing the ability to collect the fruit data required to assess alternate bearing, long juvenility period, and heavy fruit drop, etc. [117]. Nakagawa *et al*. [118] identified the FLOWERING LOCUS T-like gene and genes related to gibberellin metabolism in biennial-bearing mango trees. *FT*, *AP1*, and *LFY* genes showed upregulation in mango leaves during flower induction. Twenty-six genes were differentially expressed (DEGs) in Neelum (regular) and Dashehari (Irregular) cultivars, suggesting their roles in the alternate bearing. The DEGs belonged to hormone synthesis, alternate-bearing, and carbohydrate metabolism pathways. Furthermore, three genes (SPL-like gene, Rumani GA-20-oxidase-like gene, and LOC103420644) showed significant changes in expression patterns in both cultivars, suggesting their potential roles in alternate bearing. These findings provide initial information about the genetic basis of alternate bearing, and we surmise that multiple pathways are involved in alternate bearing [116]. Some other flowering-related genes (Table 6) have been overexpressed in Arabidopsis for functional studies. A few sporadic type studies have tried to explore the genetic basis of alternate flower bearing in mango, but detailed studies are lacking.

**Table 6:** Identification or functional analysis of flower, fruit, and stress-related candidate genes.

**Trait/Role**	**Cultivar**	**Type of study**	**Genes identified/characterized**	**Reference**
**Flowering and malformation**	Amrapali	Transcriptomic and qRT-PCR analysis	*GA20OX3*, *AGL24,* and *LDL2*	[119]
**Alternate bearing/flowering**	Neelum and Dashehari	Comparative transcriptomic and qRT-PCR analysis	*SPL-like gene*, *Rumani GA-20-oxidase-like gene*, and LOC103420644	[116]
**Alternate bearing**	Irwin	Comparative expression analysis	*MiFT*	[118]
**Flowering/alternate bearing**	Dashehari	Comparative transcriptomic analysis	Identified 85 DEGS in bearing and nonbearing tissues	[120]
**Flowering**	SiJiMi	Overexpression in Arabidopsis	*MiTFL-1*, *MiTFL1–2*, *MiTFL1–3,* and *MiTFL1–4*	[121]
**Flowering**	SiJiMi	Overexpression in Arabidopsis	MiLFY	[122]
**Flowering**	SiJiMi	Overexpression in Arabidopsis	*MiCOL1A* and *MiCOL1B*	[123]
**Flowering**	SiJiMi	Overexpression in Arabidopsis	*MiFT1*, *MiFT2*, *MiFT3*	[124]
**Flowering**	SiJiMi	Overexpression in Arabidopsis	*MiSVP1* and *MiSVP2*	[125]
**Flowering**	SiJiMi	Overexpression in Arabidopsis	*MiAP1–1* and *MiAP1–2*	[35]
**Flowering**	SiJiMi	Overexpression in Arabidopsis	*MiCOL16A* and *MiCOL16B*	[126]
**Fruit color**	Amrapali	Transcriptomic and phylogenetic analysis	*MiCHS*, *MiCHI*, and *MiF3H*	[127]
**Fruit development and ripening**	Kaituk’, ‘Nam Dok Mai No.4′, and ‘Nam Dok Mai Sithong’	Content and qRT-PCR analysis	*MiPSY*, *MiPDS*, *MiZDS*, *MiCRTISO*, *MiLCYb*, *MiLCYe*, *MiHYb*, *MiZEP*, *MiCCD1*, *MiNCED2*, and *MiNCED3*	[128]
**Fruit ripening and softening**	Alphonso	Transcriptomic and qRT-PCR analysis	38 genes of metabolic pathways	[113]
**Fruit ripening and softening**	Dashehari	Comparative Transcriptomic and qRT-PCR analysis	SAM synthase, ACC synthase, ACC oxidase, *ETR*, *EIN3*, endo beta 1–4 glucanase, expansin, pectate lyase, and polygalacturonase	[129]
**Fruit**	Dashehari	Expression analysis	*MiExpA1*	[130]
**Fruit ripening**	Kent	Transcriptomic and qRT-PCR analysis	*ACO*, *ETR1*, *ERS1*, *EIN4*, *EXP*, *AGAL*, *LCYB*, and *CHYB2*	[27]
**Disease resistance (*Colletotrichum Gloeosporioides*)**	Zill	Transcriptomic and qRT-PCR analysis	*ERFs*, *NBS-LRR*, *NPR1*, and *PR*	[131]
**Salt and drought tolerance**	SiJiMi	Overexpression in Arabidopsis	*MiPR1A*	[132]
**Salinity and drought**	Jin Huang	Overexpression in Arabidopsis	*MiCOL9A* and *MiCOL9B*	[133]
**Drought**	SiJiMi	Overexpression in Arabidopsis	*MiCOL1A* and *MiCOL1B*	[134]
**Drought**	SiJiMi	Overexpression in Arabidopsis	*MiCOL16A* and *MiCOL16B*	[126]

**Abbreviations:** qRT-PCR (Quantitative Reverse Time-Polymerase Chain Reaction), DEGS (Differentially Expressed Genes)

Mango malformation and anthracnose are the most devastating diseases of mango worldwide. The malformation is caused by different species of the fungus Fusarium, including *Fusarium mangiferae*, *F. mexicanum*, and *F. sterilihyphosum*. The susceptibility of different mango cultivars to mango malformation disease varies significantly, with conflicting reports and no experimental evidence to support them. For example, one source reported the ‘Ewais’ cultivar as moderately susceptible, but showed low and high susceptibility in other references [135]. Anthracnose appears in field and storage (pre- and postharvest stages) and causes a significant reduction in yield and fruit quality. The relationship between mango and *Colletotrichum* species (causative agent of anthracnose) is still unknown. Few mango cultivars and *Mangifera* species show resistance against anthracnose [136, 137]. No studies have been performed to identify the novel resistance genes in these genotypes for pyramiding genetic resistance in mangoes. Hong *et al*. [131] investigated the effect of borate on postharvest *Colletotrichum gloeosporioides* infection and reported some defense-related genes having a role in the anthracnose resistance mechanism. However, functional validation of the genes has not been performed. β-1,3-GLU2 has critical roles in the primary defense response of mango against anthracnose. Allele-Specific Polymerase Chain Reaction (AS-PCR) assays and phenotyping suggested that two alleles of SNP 21881933 are linked with anthracnose resistance [138]. Studies clarifying the genetic basis of mango malformation and anthracnose are not available.

## Marker selection using GWAS

Genome-wide association studies (GWAS) have been recognized as a promising approach for trait association studies in various fruit crops, including mango. GWAS offers a valuable avenue to regenerate and revise the genetic understanding of mango, enabling researchers to identify key genomic regions associated with desirable traits. By harnessing the power of GWAS, breeders can expedite the development of improved mango varieties by targeting specific genomic markers linked to traits of interest. GWAS studies in fruit crops can establish associations between genotypes and phenotypes by examining and testing variations in allelic frequencies of genetic variants. This approach enables researchers to assess the differences in allele distribution and their potential impact on observable traits [139, 140]. GWAS has greatly contributed to the identification of robust associations for various traits and diseases of interest [140]. This powerful technique has been instrumental in uncovering the underlying biology of phenotypes, estimating their heritability, and calculating genetic correlations, among other valuable insights. For example, GWAS analysis has been utilized to map the short chilling requirement, flowering time, and diverse fruit quality traits in peaches, leading to the identification of candidate genes associated with these traits [141]. Moreover, candidate genes regulating fruit ripening have been identified through GWAS in apples. Ma *et al*. [53] used genome resequencing data from 224 mango accessions and performed GWAS analysis for flowering ability, volatile compounds related to aroma, and fruit weight. Twenty-six loci linked with these parameters were identified, and 422 genes linked with identified loci were also predicted. These methods have been applied to investigate numerous traits in various fruit crops. However, there are fewer studies on mango, as it requires many samples to make associations, and difficult to obtain values in mango. Recently, GWAS analysis has been extended to include metabolomics data, known as mGWAS, which provides a more comprehensive understanding of metabolite biosynthesis. Several recent reports have demonstrated the successful application of these techniques in perennial crops. Consequently, the potential of mGWAS in studying mango crop populations appears promising and should be further explored.

## Transcriptomics-based insights into mango breeding

In the last decade, significant advancements have been made in mango breeding, such as the swift selection of parent plants and their offspring, streamlining breeding programs, and accurately assessing the genetic relationships between different mango cultivars. The implementation of modern plant breeding techniques has revolutionized mango breeding and paved the way for remarkable progress in the field [142]. Significant progress has recently been made in understanding the mechanisms behind the softening and ripening process in mango fruit cultivars. However, there is still a lack of knowledge, and advancements in omics-based biotechnological approaches, including genomics, transcriptomics, metabolomics, and proteomics, can play a crucial role in deciphering the ripening phenomena of mango fruit and understanding its postharvest physiology.

The availability of extensive transcriptomic data for different mango cultivars, developmental stages, tissues, and treatments has greatly facilitated the investigation of functional genes. Transcriptomic studies have reported genes and proteins involved in processes and pathways such as spongy tissue development [143], fruit ripening [144], aroma production [145], and disease resistance [146]. For example, the RNA-Seq analysis of red and green fruit following *C. gloeosporioides* infection on their respective red and green sides revealed that resistance in the red fruit is linked to the upregulation of the brassinosteroid, ethylene, and phenylpropanoid pathways [146]. To identify the genetic network involved in the defense response of mango fruit, the host’s reactions against *C. gloeosporioides* were examined using Illumina paired-end sequencing technology, and expression analysis of 35 defense-related genes was investigated. The study revealed that a majority of defense-related genes, e.g. nucleotide-binding site leucine-rich repeats (NBS-LRRs), ethylene response factors (ERFs), six pathogenesis-related proteins (PRs), and nonexpressor of pathogenesis-related genes (NPRs) were upregulated following pathogen infection [131]. These studies highlight the value of transcriptomic data in understanding the genetic networks underlying fruit quality, disease resistance, and overall crop productivity, and pave the way for future studies.

## Metabolomics and proteomics applications in mango research

While transcriptomics provides insight into gene-level regulation, metabolomics provides a snapshot of the biochemical outcomes, enabling a more complete understanding of fruit traits and stress responses. Regarding specific agronomical traits in mangoes, metabolomics analysis studies have explored various aspects beyond growth and development. One important quality attribute of fruits is their flavor, which encompasses taste and aroma. The volatile compounds can provide information about the aroma of a fruit. Researchers examined mango cultivars with diverse genetic backgrounds and geographical origins by identifying natural compounds and analyzing their quantitative and qualitative variations. The cultivars exhibited variations in their volatile composition, suggesting that those from different regions have been subjected to diverse selection criteria regarding aroma [147, 148]. Such studies are useful for the mango industry and breeding programs aiming to develop cultivars with specific flavors. Many ions and compounds (methanol, ethanol, acetaldehyde, and esters) linked with fruit maturity have been identified [149]. Metabolite profiling of cultivar ‘Alphonso’ and two different natural mango populations revealed a total of 66 metabolites that were unique to either mature unripe or ripened pulp tissue [150]. These studies emphasize the significance of investigating natural mango populations with diverse genetic backgrounds.

Furthermore, target-based metabolomics is an essential tool for uncovering key pathways. Suh *et al*. [151] investigated the biosynthetic mechanisms of important flavor compounds in three mango cultivars (Saigon, Glenn, and Mamme) and focused on five core metabolic pathways: phenylalanine biosynthesis and metabolism, terpenoid backbone biosynthesis, linoleic and linolenic acid route, butanoate metabolism, and carbon fixation and sucrose metabolism. A comparative transcriptome analysis and targeted metabolomics of high total soluble solids in mango cultivar ‘Tainong-1’ and the low total soluble solids in cultivar ‘Renong-1’ showed that the high sugar content in ‘Tainong-1’ is likely influenced by metabolic and biosynthetic processes, potentially regulated by transcription factors such as MYB and NAC [152].

Antioxidant compounds in the peels of high-quality elite and local mango cultivars were determined at different stages of postharvest using metabolomics-based approaches [153]. Metabolomics studies are crucial for understanding abiotic defense mechanisms. In the case of ‘Chaunsa White’ cultivar, Gas Chromatography–Mass Spectrometry (GC–MS) analysis was performed on various fruit samples to investigate metabolic pathways at different stages of development. This analysis provides valuable insights into how plants respond to abiotic stress [154]. Moreover, metabolomic fingerprinting is an essential aspect of studying the domestication history and a valuable tool for assessing metabolite differences through comparative profiling and fingerprinting analyses. For example, Farag *et al*. [155] performed a metabolomic fingerprinting study in Egypt for a better understanding of the distinct characteristics and chemical composition of Egyptian mango cultivars grown in different regions.

Proteomics can identify the proteins essential for fruit development and ripening and determine their impact on nutritional and sensory characteristics. Proteomic techniques have successfully identified numerous proteins and their associated metabolic pathways that have a role in mango fruit development, fruit coloration, maturity, ripening stages, and shelf life. For example, a mass spectrometry-based approach aligned with database searches of mango-derived expressed sequence tags and proteins led to the identification of 1001 peptides in mango leaves, which matched 538 known proteins [156]. Fruit ripening is a complex process, and comparative proteomic studies can help identify the proteins involved in maturity and ripening. In a comparative proteomic analysis, a decrease in proteins linked with hormone biosynthesis and carbon fixation during ripening was identified, and proteins involved in catabolism and stress response (oxidative stress and defense against abiotic factors and pathogens) were observed to accumulate [157]. Furthermore, 2D gel electrophoresis (2D-GE) in combination with Matrix-assisted laser desorption ionization–time of flight (MALDI-TOF/TOF) analysis was used to identify proteins with differential abundance during the ripening process of two mango varieties (Chokanan and Golden Phoenix). Proteins differentially expressed in the mango fruit mesocarp during the ripe and unripe stages were categorized into three groups: proteins involved in cell wall disintegration, ethylene synthesis, and aromatic volatiles. In addition to these categories, a fourth distinct protein category related to energy and carbohydrate metabolism was identified in the ‘Chokanan’ [158]. Liquid chromatography–Tandem mass spectrometry (LC–MS/MS) based proteomics analysis of the pericarp and pulp of ‘Zill’ mango fruit successfully identified a total of 7536 peptides that corresponded to 2754 proteins [159].

Transcriptome studies have contributed to the identification of genes involved in different processes. By integrating transcriptome and metabolome data, mango germplasm resources can be evaluated to uncover the genetic and biochemical variability linked with quality traits. The integration of metabolomics and transcriptomics will enhance mango breeding efforts. Through multiomics exploration, researchers have identified that the development of abiotic stress, particularly oxidative stress, induces disturbances in various metabolic pathways and their interconnectedness, ultimately forming spongy tissue in mangoes [143]. This has shed light on the alterations in ethylene and flavonoid biosynthesis, cell wall degradation, fruit ripening, and flavor formation, which collectively compromise the distinctive characteristics of mangoes affected by spongy tissue disorder.

## Research limitations and future prospectives

**Figure 3:**
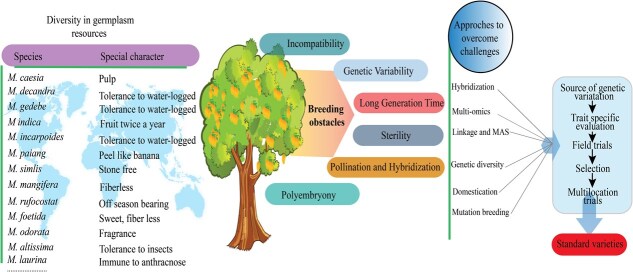
Genetic resources utilization and possible role of genetic resources in crop improvement. The figure describes the genetic diversity of *Mangifera* species, highlighting special characteristics such as soft pulp, tolerance to waterlogging, twice fruiting per year, easily removable peel, stone-free fruits, sweetness with less fiber, increased fragrance, and resistance to diseases and pests. These traits can be harnessed through strategies like grafting, hybridization, multi-omics approaches, and mutation breeding to develop superior mango cultivars with better fruit quality, high yield, adaptability to diverse climatic conditions, and resistance to diseases and pests.

**Figure 4:**
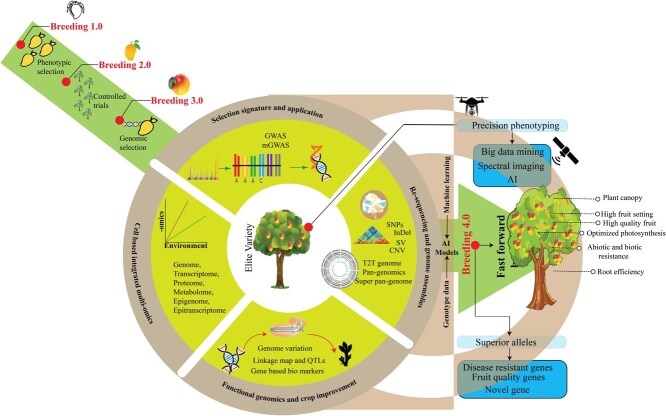
Multiple dimensional overviews of genomic and functional crop improvement strategies in mango. This figure explains the evolution of mango breeding from phenotypic selection (Breeding 1.0) and controlled trials (Breeding 2.0) to genomic selection (Breeding 3.0). It illustrates the integration of genome-wide association studies, multiomics, population genetics, and functional genomics with big data mining, spectral imaging, and AI for developing mango cultivars having high fruit quality, disease resistance, and optimized photosynthesis. **Abbreviations:** GWAS (Genome-Wide Association Studies), mGWAS (Metabolite Genome-Wide Association Study), SV (Structural variations), SNP (Single Nucleotide Polymorphism), InDels (Insertions and Deletions), CNV (Copy Number Variation), T2T (Telomere to Telomere), AI (Artificial Intelligence).

Mango holds a special position in fruit crops. Conventional breeding methods for mango improvement have been hindered by the mango tree’s lengthy juvenile phase. As a result, efforts for genetic improvement of mangoes using genomics and molecular biology-based advanced techniques have opened new possibilities, such as genome editing. These tools can significantly influence the breeding process for perennial crops. Shortening the juvenile period, the use of advanced next generation sequencing (NGS) methods, gene editing, and transfer technologies will aid in exploring the genetic potential of mango germplasm resources and provide a foundation for improvement (Figs 3 and 4).

The full potential of mango germplasm resources has not been harnessed. Nearly, 90% of the collected germplasm resources have undergone basic phenotypic trait evaluation. Similarly, 30%–50% and < 10% of germplasm resources have been evaluated for physicochemical traits and genotype description, respectively. Therefore, many germplasm resources lack accurate identification, resulting in insufficient excavation of excellent traits and key genes. These factors collectively hinder the mango breeding progress. A strategy for harnessing the potential of diverse germplasm resources has been described in Fig. 3.

The figure describes the genetic diversity of *Mangifera* species, highlighting special characteristics such as soft pulp, tolerance to waterlogging, twice fruiting per year, easily removable peel, stone-free fruits, sweetness with less fiber, increased fragrance, and resistance to diseases and pests. These traits can be harnessed through strategies like grafting, hybridization, multiomics approaches, and mutation breeding to develop superior mango cultivars with better fruit quality, high yield, adaptability to diverse climatic conditions, and resistance to diseases and pests.

Advancements in single-cell multiomics technologies can speed up the evaluation of germplasm potential by investigating the complex molecular makeup of mangoes. These methods have revolutionized research in molecular cell biology. However, progress in mango research has not been on par with these advancements. Integrated omics approaches are necessary for understanding mango plants’ temporal and spatial changes under various stresses. However, there is a lack of epigenomic and functional genomic studies in mangoes. The recently developed haplotype-resolved T2T genome now enables more precise genetic studies, but challenges remain in tissue culture and genetic transformation that hinder functional validation. The development of a comprehensive pangenome will be essential to facilitate genetic diversity studies and marker-assisted breeding in future mango improvement programs. Similarly, understanding epigenetic changes and functional validation are necessary for understanding the response of genes to different stresses. The use of functional genomics with other omics disciplines will help to uncover the connections between various genomes and phenotypes under specific stress conditions. However, single-cell integrated multiomics methods face challenges, such as high sequencing costs and limited cell coverage [160]. Overcoming these challenges will facilitate the development of sophisticated strategies and the creation of comprehensive atlases that integrate multiple omics datasets and different timescales, thereby increasing our understanding of mango genetics and mango breeding efforts.

In fruit plants, a long juvenile period also hinders progress efforts and can be reduced by maintaining environmental conditions that negatively affect plant vigor [161, 162]. In apples, manipulation of the environmental (optimal or controlled) conditions shortened the juvenility period from 5 years to 10 months [163]. Moreover, direct transgenic expression of flowering time genes, like *FT*, has induced early flowering in various woody perennial species, such as citrus and plum, where constitutive *FT* expression drastically shortened the juvenile phase and generation time [164, 165]. Although information regarding alteration in the juvenile period in mango is not available in the literature, the juvenile period can be reduced by optimizing growth conditions and modulating signal transduction pathways for flowering. For example, the use of transgenic rootstock expressing FT can promote early flowering. Integrating these approaches with genomic prediction can significantly shorten the juvenility period and accelerate cultivar development.

Genomic prediction allows to skip some early evaluation steps, thereby reducing the large-scale phenotyping, resources, and cost, and shortening the breeding cycle [166, 167]. For example, Sun *et al*. [168] predicted high breeding values of pear at an early stage using eight fruit traits. In apples, accessions with a favorable fruit texture were predicted [169]. Brault *et al*. [170] compared two ongoing grape breeding programs to identify factors influencing the prediction accuracy. For the first time, they used a multitrait selection index to select superior genotypes. This study provided an avenue for integrating genomic prediction into grapevine breeding programs. Compared to other fruit crops (grapes, apple, and citrus), the mango genome has been less extensively studied, and advanced methods have not been widely used in mango breeding. However, the availability of high-quality mango genomes paves the way for the integration of genomic prediction in mango breeding programs to overcome the breeding obstacles, notably the long juvenile period.

Breeding efforts can progress rapidly by accurately adopting genome editing techniques, as efficient genome editing has been carried out in different horticultural crops for crop improvement [171, 172]. However, no progress has been made in mangoes due to the lack of efficient regeneration and genetic transformation systems. The gene transformation system has not been developed due to the late availability of the reference genome. The availability of mango genomes has paved the way for developing genetic transformation and genome editing protocols. Successful development of these protocols will facilitate mango breeding programs. Integration of genomic prediction with genome editing can revolutionize mango breeding efforts.

Conventional plant breeding has reached its limitations in sustaining the continuously expanding global population and alleviating the impact of global climate change [173]. Based on the methods and techniques used, scientists have divided breeding into four generations. Initially, people relied on phenotypic selection but lacked professional breeding technologies (Breeding 1.0). Later, with some advancements, breeders introduced purposeful strategies with Mendelian genetics (Breeding 2.0). Advancements in techniques and sequencing technologies enabled integration of molecular markers and genomic data for trait selection (Breeding 3.0) [174]. Recently, some crops, like potato, have achieved Breeding 4.0 generation (identified and deleted deleterious alleles–hybrid potato genome design), while others, like tomato and rice, are expected to achieve it soon [97, 175]. Unfortunately, mango breeding is still lagging due to the long juvenile period, high heterozygosity, and complex genome. To overcome these challenges, we need to learn and adopt techniques used in potato genome design.

The precise estimation of genomic breeding values is crucial in genomic prediction and requires the analysis of thousands of markers across the genome [176]. With the use of machine learning tools, genomic prediction will be easier, and the breeding cycle can be shortened—the main obstacle of mango breeding. Moreover, integrating Interactome Big Data (an effective approach for representing interactions within an organism) with machine learning can identify new functional genes based on already known genes. This integrative strategy has advantages over forward and reverse genetics approaches, as it eliminates the need to create multiple large populations and undergo stable transformation. The required data can be retrieved from public databases, so this is a cost- and time-effective approach [177]. Due to the advancements in sequencing technologies and assembly methods, the publicly available omics data for mango is increasing. Integration of Interactome Big Data with machine learning can fuel the breeding progress of mangoes (Fig. 4). In this way, mango’s main breeding obstacles can be addressed (long breeding cycle and generation of large populations).

## Concluding remarks

During the last decade, significant progress has been made in mango genomic studies, which has provided a platform for improvement in breeding practices. These advancements offer important resources for mango crop improvement and domestication studies with a prime focus on QTLs linked with disease resistance, fruit quality, and shelf life. Importantly, getting insights into the domestication and genetic improvement of a crop at the whole-genome level can greatly contribute to future endeavors aimed at enhancing yield and other desirable traits. A comprehensive pangenome needs to be built using both cultivars and wild genomes for a better understanding and improvement of mango. This will be particularly valuable for addressing genotype–environment interactions and identifying climate-resilient traits. Additionally, functional validation of mango QTLs identified in GWAS will help to understand the genetic basis of complex agronomic traits. However, progress in this area remains limited by challenges in tissue culture and genetic transformation systems. Traditional mango breeding is time-consuming and may require decades to develop a desirable variety. Modern phenomics, genomics, synthetic biology tools, and machine learning can accelerate the mango breeding process through gene validation and precision genome editing. Future efforts should focus on integrating these advanced technologies while developing solutions for the current limitations in transformation protocols and multienvironment testing.
